# Psychometric properties of the maternal breastfeeding evaluation scale: a confirmatory factor analysis

**DOI:** 10.1186/s12884-024-06693-8

**Published:** 2024-07-18

**Authors:** Silvia Escribano, Raquel Herrero-Oliver, Antonio Oliver-Roig, Miguel Richart-Martínez

**Affiliations:** 1 Department of Nursing, Faculty of Health Sciences, University of Alicante, Carretera San Vicente del Raspeig s/n, Alicante, 03690 Spain; 2 Dr. Balmis University General Hospital, Alicante, Spain

**Keywords:** Breastfeeding experience, Maternal, Psychometrics, Satisfaction, Spanish version

## Abstract

**Background:**

It has been suggested that maternal satisfaction should be included as an additional and appropriate outcome indicator in relation to the breastfeeding process. The aim of this study was to analyze the psychometric properties of various existing versions of the Maternal Breastfeeding Evaluation Scale in a Spanish sample.

**Methods:**

This was a longitudinal observational study, evaluated at three different time points: in the hospital after delivery, and then at five and 12 months after delivery in a Spanish sample. A total of 690 mother participated in this study.

**Results:**

Confirmatory factor analysis results indicated an improved fit of the data to the original model (CFI = 0.984; TLI = 0.982; RMSEA = 0.079). All dimensions of the Maternal Breastfeeding Evaluation Scale are positively associated with breastfeeding rates and negatively associated with perceived difficulty in continuing to breastfeed after returning to work at five months postpartum. Moreover, the scale can predict breastfeeding behavior at 12 months postpartum.

**Conclusions:**

The results of this study indicate that the structure of the original version of the Maternal Breastfeeding Evaluation Scale mean it is a is valid and reliable tool for assessing maternal perceptions of the breastfeeding experience in Spain. This research enhances our understanding of maternal satisfaction with the breastfeeding experience and its potential implications for supporting breastfeeding practices. It is an opportunity for the academic, healthcare, and policy sectors to develop more effective interventions to improve breastfeeding rates and ensure a positive experience for mothers.

**Supplementary Information:**

The online version contains supplementary material available at 10.1186/s12884-024-06693-8.

## Introduction

Breastfeeding is the only feeding method with a unique nutritional composition capable of adapting to the baby’s needs [1]. It is considered the best source of nutrition during the first years of life, and the benefits for both mother and infant have been well-documented in the literature [1]. For example, breastfeeding is known to protect children against common infections [2], reduce their risk of cancer [3], and improve their cognitive development [4]. Furthermore, exclusive breastfeeding has been linked to postpartum weight loss in mothers [5], as well as lower rates of ovarian and breast cancer, and reduced risk of heart disease, among other benefits [6]. Despite recommendations for exclusive breastfeeding during the first six months, supplemented with other appropriate foods until the age of two or over [1], the rates of exclusively breastfed children at six months in Europe vary from 13 to 39% [7]. In Spain in particular, the rate of exclusive breastfeeding at six months is reported to be 28% [7].

It is therefore essential to identify factors associated with breastfeeding—especially those that can be modified— to develop effective interventions [8, 9]. The premature cessation of breastfeeding is negatively influenced by an unfavorable legislative, employment, and social environment, as well as professional activities that create obstacles to initiating and continuing breastfeeding [10]. A recent systematic review [11] found that the cessation of breastfeeding before six months is associated with personal sociodemographic factors (such as young maternal age, low maternal educational level, returning to work, and primiparity), physical factors (such as perceived or actual inadequate milk supply and sore or painful nipples), and psychosocial factors (such as experiencing symptoms of depression). The decision of mothers to discontinue breastfeeding is therefore a complex and multifactorial phenomenon [12], supporting the view expressed by Leff et al. [13]: “The women described successful breastfeeding as a complex interactive process resulting in mutual satisfaction of maternal and infant needs.”

However, breastfeeding success has traditionally been measured in terms of its duration or the absence of problems, without taking into account maternal perspectives and experiences, including satisfaction with breastfeeding [14, 15]. Satisfaction with the lactation experience is considered a relevant outcome measure [16], as it is vital to enhancing our understanding of the aspects deemed to be important by breastfeeding mothers. This understanding is essential for effectively promoting successful breastfeeding practices beyond the mere duration of breastfeeding [13]. In any event, greater satisfaction with breastfeeding has been associated with improved outcomes in breastfeeding rates, such as exclusive breastfeeding up to six months [10], and a reduction in the discontinuation of exclusive breastfeeding before six months [8]. In an Australian cohort, breastfeeding satisfaction was a stronger predictor of discontinuation than breastfeeding problems [17]. Similarly, in a Polish study, the authors concluded that maternal satisfaction with breastfeeding was among the predictors of exclusive breastfeeding after six months postpartum [18].

## Background

The Maternal Breastfeeding Evaluation Scale (MBFES) [13] is a unique, valid, and reliable tool [13, 19] that assesses various aspects of the maternal breastfeeding experience across three dimensions: “Maternal enjoyment/Role attainment,” which reflects positive feelings about the physical and emotional aspects of the breastfeeding experience; “Infant Satisfaction/Growth,” which is primarily related to the infant’s weight gain and growth, and their emotional responses to nursing; and “Lifestyle/Maternal Body Image,” which is related to breastfeeding as a burden on other areas or activities. This scale originated from previous qualitative research exploring maternal descriptions of successful breastfeeding experiences [20].

The MBFES scale has been used in several studies [8, 14, 17, 21, 22] and has been cross-culturally adapted and validated in several languages, including Portuguese [23], Arabic [16], Japanese [24], and Spanish [25]. All adapted versions have undergone a new exploratory factor analysis, demonstrating adequate psychometric properties and producing very similar versions. However, compared to the proposed original structure (Appendix 2), all versions include minor modifications and item restructuring. For example, the Japanese version (JMBFES) [24] is the shortest among the various validations, probably because a more restrictive criterion was used in the exploratory factor analysis, eliminating all items with factor loadings < 0.50. The JMBFES [24] is made up of 23 items and three dimensions: Maternal Satisfaction (11 items), which aligns with the original version; “Potentially Negative Aspects” (5 items), corresponding to the Lifestyle/Maternal Body Image dimension of the original version; and “Perceived Benefit to Baby” (7 items), equivalent to the Infant Satisfaction/Growth dimension. However, to date, no confirmatory analysis has yet been conducted on the different versions obtained to assess model fit. This is necessary to confirm the factor analysis [13] and determine which version performs better.

In terms of validity criteria, none of the studies [13, 16, 23, 24], including the Spanish version [25], have examined the predictive ability of the scale in relation to breastfeeding. Nabulsi et al. [16] is the only study to have used correlations to analyze the associations between the scores and exclusive breastfeeding at three months postpartum. For practitioners, managers, and clinical decision makers alike, having a detailed understanding of the clinical utility of the scale in relation to breastfeeding success may be useful.

### Purpose

The aim of this study was therefore to analyze the psychometric properties of various existing versions of the Maternal Breastfeeding Evaluation Scale in a Spanish sample.

We put forward three main hypotheses. Hypothesis 1: that the data will show an adequate fit to the original version [13], established through rigorous content validity analysis [20]. Hypothesis 2: in terms of validity, that higher scores on the dimensions of the MBFES will be positively associated with overall satisfaction with breastfeeding. Hypothesis 3: in addition, that higher scores on the dimensions of the MBFES will be negatively associated with difficulties in continuing to breastfeed after returning to work. A recent systematic review highlighted the association between returning to work and cessation of breastfeeding [11]. Hypothesis 4: in terms of criterion validity, that the three dimensions of the MBFES scale will be capable of predicting breastfeeding at 12 months postpartum. This is in accordance with the literature that has shown an association between MBFES and breastfeeding rates [8, 10, 18].

## Methods

### Design and participants

This was a longitudinal observational study, evaluating participants at three distinct time points: in the hospital after delivery, and then at five and 12 months after delivery.

The data in this article correspond to a wider study investigating factors associated with breastfeeding discontinuation, which involved women who were recruited during pregnancy from six hospitals in eastern Spain during the period 2010–2011. These hospitals were undergoing improvement processes linked to the World Health Organization’s Baby-Friendly Hospital Initiative, and were in need of a tool capable of assessing more than just breastfeeding rates. Pregnant mothers participating in the study were recruited during a third trimester check-up at either the midwife’s primary care clinic or the fetal physiopathology unit of the attending hospitals, between 28 and 42 weeks of pregnancy. A total of 1,354 mothers initially agreed to participate in the project at baseline, with 1,122 meeting the inclusion criteria. A final convenience sample of 690 (61.50%) postpartum breastfeeding women completed a postal questionnaire five months after delivery, providing the outcome data for this study (see Appendix 1).

We analyzed whether participant attrition at five months was related to sociodemographic variables. We observed that non-responders were younger (M = 30.98; SD = 5.28) than those who responded (M = 32.26; SD = 4.41) (t=-4.34; *p* < 0.001). We also found that the attrition rate increased gradually with decreasing socioeconomic status (10% >€60,000 to 57.7% <€6,000; chi-square = 44.63; *p* < 0.001) and decreasing educational level (from 25% of those with a university education to 100% of those who had not completed primary education; chi-square = 80.04; *p* < 0.001). We found a relationship between attrition and the marital status variable, with single women responding less (52.8%) than those who were married or in a civil partnership (chi-square = 8.57; *p* < 0.05). No relationship was found between attrition and having previous children (V of Cramer = 0.05; *p* = 0.12).

To be included in this research study, participants had to able to read and speak Spanish. Mothers were excluded if they: (a) had not initiated any type of breastfeeding (exclusively or partially) at discharge, (b) had a preterm delivery, multiple births, or medical conditions that prevented or significantly impeded breastfeeding, including maternal issues such as HIV infection or previous breast surgery, or neonate issues such as an Apgar score below six at five minutes of life, neonatal sepsis, cleft lip, cleft palate, or Down syndrome.

### Sample description

A total of 690 participants satisfied the inclusion criterion and completed the MBFES scale at five months postpartum (see Appendix 1). The mean age of the women was 32.20 (SD = 4.39) and 93.6% (*n* = 646) of the sample were Spanish. In terms of marital status, 89.9% (*n* = 620) were married or in a civil partnership, while the remainder were single or separated/divorced. However, only seven mothers (1%) reported not living with their current partner. With regard to socioeconomic data, 15.9% (*n* = 110) had an annual income below €8,999, 31.8% (*n* = 219) from €9,000 to €17,999, 41.6% (*n* = 287) from €18,000 to €44,999, and 3.9% (*n* = 27) over €45,000. With respect to educational level, 61.5% (*n* = 424) reported having at least a high school education. As regards motherhood, 57% of the women were first-time mothers (see Table 1).

**Table 1:** Sociodemographic variables (*N* = 690)

		*n* (%)
Marital status, n (%)		
	Married or civil partnership	620 (89.9)
	Separated or divorced	17 (2.5)
	Single	51 (7.4)
	Missing	2 (0.3)
Nationality, n (%)		
	Spanish	646 (93.6)
	Not Spanish	44 (6.4)
Annual income, n (%)		
	< €6000	54 (7.8)
	€6,000–€8,999	56 (8.1)
	€9,000–€11,999	68 (9.9)
	€12,000–€17,999	151 (21.9)
	€18,000–€29,999	186 (27)
	€30,000–€44,999	101 (14.6)
	€45,000–€60,000	18 (2.6)
	> €60,000	9 (1.3)
	Missing	47 (6.8)
Educational level, n (%)		
	Incomplete primary education	66 (9.6)
	Primary education	198 (28.7)
	High school education	151 (21.9)
	Higher education (undergraduate)	273 (39.6)
	Missing	2 (0.3)
Number of births		
	Primiparous	393 (57)
	Multiparous	297 (43)

With respect to the variables collected at the time of hospital discharge after delivery, 62% (*n* = 428) of the women had a vaginal birth, 20.6% (*n* = 140) had a cesarean, and data for this variable was missing in 17.4% (*n* = 120) of the cases.

In terms of breastfeeding, 100% of the mothers were providing some form of breastfeeding at the time of discharge. Of these, 59.1% (*n* = 408) were exclusively breastfeeding, 18.1% (*n* = 125) were partially breastfeeding, and specific information about the type of breastfeeding at the time of discharge was not available in 22.8% (*n* = 157) of the cases.

### Instruments

A self-administered questionnaire was developed to collect data on sociodemographic variables as well as factors associated with breastfeeding and childbirth. The sociodemographic variables collected during pregnancy were: age, nationality (Spanish/other), marital status (married or civil partnership, separated or divorced, single, widowed), cohabitation with current partner (yes/no), educational level (incomplete primary education, primary education, high school education, higher education), annual family income (on a seven-point scale ranging from less than €6,000 to more than €60,000), and number of births (primiparous or multiparous).

The variables collected at postpartum hospital discharge were type of delivery (vaginal birth/cesarean) and type of breastfeeding (exclusive/partial). The different types of breastfeeding were defined as follows: exclusive breastfeeding (EBF), where infants receive only breast milk, with the possible exception of rehydration solutions, drops, and syrups; and partial breastfeeding (PBF), where infants receive any amount of breast milk, with or without other liquids or foods.

The variables collected at five months were: type of breastfeeding (1 = any breastfeeding, 2 = no breastfeeding), with “any breastfeeding” (ABF) being defined as the baby receiving any amount of breast milk; return to work (dichotomous, yes/no); perceived difficulty in continuing to breastfeed due to return to work (0 = no difficulty to 100 = highest difficulty); perception of the breastfeeding experience using the Maternal Breastfeeding Evaluation Scale (MBFES) [13]; and satisfaction with breastfeeding based on the question “On a scale from 0 to 100, how satisfied do you feel with how everything related to breastfeeding has gone?” (0 = not satisfied at all to 100 = completely satisfied).

The MBFES is a tool that assesses maternal perceptions of the breastfeeding experience. It consists of 30 items and three dimensions: Maternal Enjoyment/Role Attainment, Infant Satisfaction/Growth, and Lifestyle/Maternal Body Image. Previous studies have shown the English version to have adequate psychometric properties when validated in an American sample, with internal consistencies for each of the dimensions ranging from 0.91 to 0.93, 0.83 to 0.88, and 0.80 to 0.84, respectively [13, 19]. It uses a five-point Likert-type scale, ranging from “1 = Completely Disagree” to “5 = Completely Agree.” Higher scores indicate a better perception of the breastfeeding experience. Reverse-scoring was used where appropriate to ensure that higher scores were indicative of a more positive evaluation. For our study, items from the version culturally adapted to the Spanish context were used [25]. Appendix 2 provides a description of the various versions and validations of the MBFES.

Finally, at 12 months postpartum, participants were asked again if they were still breastfeeding using a dichotomous (yes/no) question.

### Procedure

Data were collected during the third trimester of pregnancy (28–42 weeks) using a self-administered paper questionnaire to capture the sociodemographic variables. Between the second and fifth day postpartum, while still hospitalized and prior to discharge, participants completed a paper questionnaire concerning the type of delivery and breastfeeding. At five months postpartum, a follow-up was conducted using a paper questionnaire sent by postal mail, again focusing on variables related to breastfeeding. Mothers answered the questionnaire within a time frame ranging from five to nine months postpartum (M = 5.39; SD = 0.87), allowing sufficient time for postal deliveries and responses. Non-respondents were sent the questionnaire up to three times, at three weekly intervals, and were provided with the required form, a pen, and a stamped, self-addressed envelope to return the responses to the research team. Finally, at 12 months postpartum, a telephone survey was conducted with mothers who were still engaged in any form of breastfeeding with their child at the time of the five-month survey to gather information on breastfeeding status.

This study followed the criteria established by the Declaration of Helsinki and the European Union Standards of Good Clinical Practice and received institutional approval from Clinical Research Ethics Committee of the Hospital “Verge dels Lliris” of Alcoy (Alicante, Spain). All the women recruited agreed to participate in the study and provided written informed consent. They were given information about the purpose of the study and how their data would be handled by the researchers. With regard to the handling and use of the collected data, only the research team members had access to the database generated, where personal data were encrypted with a unique code for each participant to minimize the use of identifiable information. Identification data were stored in a separate database, accessible only to the lead investigator. Mothers were not compensated for their participation.

### Data analysis

The programs R (version 3.4.0) and SPSS (version 26.0) were used to perform the different statistical analyses. Descriptive analyses of the sociodemographic and lactation-related variables were carried out. We calculated the mean duration and proportion of infants by age for exclusive and any breastfeeding using the survival table method, with the information collected at the time of hospital discharge, five months, and 12 months postpartum.

We included all cases where the MBFES items had been completed in full. We calculated the mean and standard deviation of the item scores and determined the proportion of respondents with the lowest or highest possible score to assess floor and ceiling effects. We used the COSMIN checklist [26] to ensure that our study met the highest quality standards. Confirmatory factor analysis was performed for each version of the MBFES to examine the structure of each one. The lavaan package in R [27] was used to perform the confirmatory factor analyses (CFA) using the weighted least squares means and variance (WLSMV) adjusted estimation method, which is appropriate for categorical variables [28]. To assess the fit of the data to the model, we calculated the Tucker-Lewis Index (TLI), the comparative fit index (CFI), and the root mean squared error of approximation (RMSEA). CFI and TLI values > 0.9 indicate an acceptable fit and values of > 0.95 indicate a good fit [29]. RMSEA values < 0.05 are considered adequate [30]. The internal consistency was analyzed using the ordinal alpha suggested for ordinal data, by calculating polychoric correlations [31].

Construct validity (hypothesis testing) was calculated using variables such as satisfaction with breastfeeding, continuation of breastfeeding, and difficulty maintaining breastfeeding upon returning to work. For quantitative variables, Pearson correlations were analyzed, and for categorical variables, a comparison of means was performed using Student’s t-test.

Finally, criterion validity was analyzed for the breastfeeding at five months postpartum variable by comparing means using Student’s t-test, and predictive criterion validity at 360 days (12 months) was calculated using binary logistic regression. The level of statistical significance was set at *p* = 0.05.

The sample size of 690 satisfied the recommendations of various criteria outlined in the literature, as summarized in Vargas and Mora-Esquivel [32]. For instance, Hair [33] recommends a minimum sample size of 200 subjects to ensure model stability. Furthermore, Catena et al. [34] suggest between eight participants for each of the latent and observed variables (33 * 8 = 264) or 15 participants for each observed variable (30*15 = 450). Finally, in line with the procedure recommended by MacCallum et al. [35], which is based on the overall fit of the model as measured by the RMSEA index, we obtained a statistical power of 0.82. The calculation was performed using Preacher and Coffman’s online software [36] with the following model parameters: lower bound of RMSEA = 0.076, upper bound of RMSEA = 0.083, and df = 402.

## Results

### Construct validity: structural validity

Based on the results shown in Appendix 3, the data fit both the original version (MBFES) [13] and the proposed Spanish version (MBFES-E) (Piñeiro-Albero et al., 2022) without the need for modification index analysis. The fit indexes for the original model were as follows: CFI = 0.984; TLI = 0.982; RMSEA = 0.079 (95%CI = 0.076–0.083). The fit indexes for the MBFES-E structure were as follows: CFI = 0.979; TLI = 0.978; RMSEA = 0.087 (95%CI = 0.084–0.091). An improved fit, particularly in the RMSEA index, is observed for the original MBFES structure compared to the MBFES-E. Factor loadings for the MBFES range from 0.51 to 0.94, except for two items with loadings lower than 0.30, i15 (λ = 0.25) and i22 (λ = 0.25). Similarly, factor loadings for the MBFES-E range from 0.54 to 0.96, with items 15 and 22 also exhibiting similar loadings: i15 (λ = 0.24) and i22 (λ = 0.26).

In terms of the structure of the short Japanese version (Hongo et al., 2013), the data also seem to fit the model with good fit indexes (Table 2). However, closer examination of the other parameters reveals a negative error variance in item 29 and an over-saturation (> 1) (Heywood case), which invalidates the model.

**Table 2:** Confirmatory factor analysis of various versions of the MBFES scale (*n* = 690)

	Chi-square	df	*p*	CFI	TLI	RMSEA	95%CI	Factor loadings
**MBFES**	2148.886	402	0.000	0.984	0.982	0.079	0.076–0.083	0.25–0.94
**MBFES-E**	2518.426	402	0.000	0.979	0.978	0.087	0.084–0.091	0.24–0.96
**JMBFES**	1363.449	227	0.000	0.987	0.985	0.085	0.081–0.090	0.25–1.02

Note: MBFES = English version; MBFES-E = Spanish version; JMBFES = Japanese version; df = degree of freedom; p = p value; CFI = comparative fit index; TLI = Tucker-Lewis Index; RMSEA = root mean squared error of approximation; CI = Coefficient Interval

### Internal consistency

With respect to the structure of the original MBFES scale, ordinal alpha values of 0.96, 0.88, and 0.82 indicate internal consistency was obtained for the dimensions of Maternal Enjoyment/Role Attainment, Infant Satisfaction/Growth, and Lifestyle/Maternal Body Image, respectively. For the Spanish version of the MBFES-E, the internal consistencies were 0.96, 0.85, and 0.82 for the same dimensions, respectively. In terms of the structure of the short version of the JMBFES, internal consistency values were 0.96, 0.90, and 0.77 for the dimensions of Maternal Satisfaction, Perceived Benefit to Baby, and Potentially Negative Aspects, respectively.

### Construct validity: hypothesis testing

Positive correlations were found between overall satisfaction with breastfeeding at five months and the dimensions of the original MBFES (*r* = 0.73; *p* < 0.001): Maternal Enjoyment/Role Attainment (*r* = 0.68; *p* < 0.001), Infant Satisfaction/Growth (*r* = 0.69; *p* < 0.001), and Lifestyle/Maternal Body Image (*r* = 0.34; *p* < 0.001).

The rate of mothers returning to work by the five-month point was 37.4% (*n* = 258). There was an inverse association between difficulty in continuing to breastfeed upon returning to work and the scores of all three dimensions of the MBFES: Maternal Enjoyment/Role Attainment (*r*=-0.30; *p* = 0.001), Infant Satisfaction/Growth (*r*=-0.23; *p* < 0.001), and Lifestyle/Maternal Body Image (*r*=-0.42; *p* < 0.001).

### Criterion validity

At the time of the five-month survey, 69% (*n* = 476) were breastfeeding, while 28.8% (*n* = 199) were not breastfeeding at all. Mothers who were breastfeeding at the time of the survey scored higher on the MBFES dimensions than those who were not breastfeeding (see Table 3).

**Table 3:** Comparison of MBFES scores and breastfeeding at five months

	ABF	No breastfeeding			
	M (SD)	M(SD)	Student’s t	df	*p*-value
**Maternal Enjoyment/Role Attainment**	72.17 (7.38)	62.47 (13.76)	9.40	246.98	**0.000**
**Infant Satisfaction/Growth**	32.94 (4.37)	26.73 (7.29)	11.20	259.65	**0.000**
**Lifestyle/Maternal Body Image**	23.09 (4.75)	21.77 (4.77)	3.28	673	**0.001**

Note: df = Degree of freedom; ABF= “any breastfeeding”, defined as when a baby received any amount of breast milk

At 12 months, 483 participants (70%) were successfully contacted, of whom 29.6% (*n* = 143/483) were still breastfeeding. The results indicate that higher scores on the MBFES dimensions of Maternal Enjoyment/Role Attainment and Infant Satisfaction/Growth predict breastfeeding at 12 months postpartum: (B=-0.058; Exp (β) = 0.94 [95% CI = 0.91–0.98]; *p* < 0.001) and (B=-0.092; Exp (β) = 0.91 [95% CI = 0.87–0.96]; *p* < 0.001), respectively. However, the Lifestyle/Maternal Body Image dimension is not a predictor of breastfeeding at 12 months (B=-0.007; Exp (β) = 0.99 [95% CI = 0.94–1.04]; *p* = 0.791).

## Discussion

In this study, we analyzed the psychometric properties of various existing versions of the Maternal Breastfeeding Evaluation Scale, which assesses maternal perceptions of the breastfeeding experience, in a Spanish context. Our first hypothesis was that the data would show an adequate fit to the original version. Confirmatory factor analysis showed that the data fit both the original structure [13] and the proposed structure for the validated Spanish version [25]. Following exploratory factor analysis [13, 25], both versions resulted in a scale made up of 30 items and three interrelated dimensions. However, in terms of dimensional structure, slight variations across the different versions have been found. The results reflected an improved fit of the data to the original model [13]. Furthermore, analysis of the abbreviated 26-item Japanese version revealed an invalid factorial solution for these data, as indicated by the observation of a Heywood case [37]. We therefore decided to use the original structure in the present study. The results also suggest that the dimensions have good internal consistency across different versions [13, 23, 25].

Second, we hypothesized that higher scores on the dimensions of the MBFES would be positively associated with overall satisfaction with breastfeeding. We found that the Maternal Enjoyment/Role Attainment and Infant Satisfaction/Growth dimensions of the MBFES scale were strongly and positively associated with overall maternal satisfaction with breastfeeding. They also show a moderate positive association with the Lifestyle/Maternal Body Image dimension. These are also in line with the findings in the original version [13]. This is precisely why Leff et al. [13] concluded that the scale is a comprehensive measure of the breastfeeding experience that goes beyond maternal satisfaction with breastfeeding. The availability of valid and reliable self-administered scales that are capable of evaluating aspects of the breastfeeding experience and satisfaction, taking into account potentially modifiable factors, is of great value [9].

Nowadays, it is important not to overlook the significant value provided by Patient-Reported Outcome Measures (PROMs) in global healthcare services. PROMs assess individuals’ perceptions of their own situation, while Patient-Reported Experience Measures (PREMs) focus on the clinical care received [38]. Both measures focus on the person-centered care paradigm [39], which prioritizes capturing the experiences and outcomes that matter to individuals over clinical outcome measures [40, 41]. As is the case in other areas of health care, there has been a growing interest in PROMs and PREMs in maternity, pregnancy, and childbirth care [42]. In this context, maternal satisfaction with the breastfeeding process, which is directly linked to PROMs, should be included as an additional and appropriate outcome indicator. Such an approach to evaluating satisfaction with breastfeeding would help professionals focus on providing care that is centered on the quality of mothers’ experiences, identifying needs and potential areas of improvement in the care they provide [13–15] during the breastfeeding process, which can then be incorporated into interventions.

Third, the results obtained were in line with the original hypothesis that higher scores on the dimensions of the MBFES would be negatively associated with difficulty maintaining breastfeeding upon returning to work. This means that mothers who perceived greater difficulty in breastfeeding after returning to work expressed lower satisfaction with their maternal role, their baby’s development, and their body image. The results highlight the challenges faced when combining breastfeeding and employment. As contextualized by Senna et al. [14] the maternal satisfaction with breastfeeding variable has been relatively unexplored, with even fewer studies exploring associated factors. Satisfaction with breastfeeding has been linked to various factors such as skin-to-skin contact or encouraging breastfeeding in the maternity ward [43], the absence of breastfeeding-related issues [17], and spousal support [14], among others. However, to the best of our knowledge, this is the first time the employment variable has been associated with breastfeeding satisfaction. As noted in the systematic review by Vilar-Compte et al. [44], existing studies are limited to assessing the impact of employment on breastfeeding rates and duration. The relationship identified between difficulty in breastfeeding after returning to work and satisfaction underscores the need for policies aimed at reducing the obstacles and challenges women face in continuing to breastfeed [44]. Tackling these issues could be pivotal in promoting a more positive breastfeeding experience for women.

Finally, we hypothesized that the MBFES scale would be able to predict breastfeeding behavior at 12 months postpartum. We found that higher scores on the Maternal Enjoyment/Role Attainment and Infant Satisfaction/Growth dimensions of the MBFES were predictive of breastfeeding at 12 months postpartum. However, no such results were found for the Lifestyle/Maternal Body Image dimension. In conclusion, the MBFES emerges as a tool capable of predicting the continuation of breastfeeding in the long term, particularly the dimensions that assess emotional aspects of the mother together with perceptions of the child’s well-being. This is in line with other studies that have pointed to these aspects, such as factors associated with the cessation of breastfeeding [11, 44].

To our knowledge, this is the first study to analyze the long-term predictive capacity of this instrument. The validated versions typically analyzed concurrent criterion validity. We also found correlations with breastfeeding at five months, meaning that mothers who continued to breastfeed scored higher in Maternal Enjoyment/Role Attainment, Infant Satisfaction/Growth, and Lifestyle/Maternal Body Image than mothers who no longer breastfed, in line with other validation studies, such as the Japanese [24], Arabic [16], and Portuguese versions [23]. Nabulsi et al. [16] observed that mothers who exclusively breastfed at one and three months scored higher in the Infant Satisfaction/Growth and Maternal Enjoyment/Role Attainment dimensions compared to those who partially breastfed, while mothers who fed their babies formula scored lower. This would therefore appear to be a consistent result irrespective of the context analyzed.

In terms of limitations of this study, we should first consider that the results reported in this study were within the context of a Spanish sample. For future lines of research, we recommend that the proposed models and structures of the different versions be validated in other samples with different characteristics to ensure structure stability. Second, the overall completion rate for mothers who filled out the questionnaire at five months was 60.50%. There were differences in the characteristics of those who dropped out of the study and those who were ultimately included. The participants who responded were more likely to be Spanish, older, and have higher levels of both education and family income. Therefore, in future studies, the instrument should also be evaluated in populations at a higher risk of attrition. Third, it is important to note that the data used for this study were collected more than five years ago. This fact should be considered when interpreting the timeliness and relevance of the results in the current context. Fourth, delays in returning the questionnaires resulted in a wider range of response times, spanning from five months up to nine months. Some of the women who responded after nine months, if they were no longer breastfeeding, answered the MBFES questionnaire based on their recollection of breastfeeding, which could introduce recall bias in these cases. Fifth, common method bias is highlighted as a potential concern for the present study as a precautionary note for studies where data have been collected using the same response method, in this case a self-administered survey [45]. However, there are a number of procedural controls for common method bias that we wish to draw attention to. For instance, the survey was designed to provide clear instructions, ensure the anonymity of responses, avoid complex items, and control methodological separation (separation of the measures in the survey) [45]. Finally, there are psychometric properties recommended for instrument validation [26] that have not been analyzed in this present paper. It would therefore be appropriate to analyze other psychometric properties of the scale, such as responsiveness and reliability, particularly test-retest reliability, in order to provide evidence of all the properties of validity and reliability.

## Conclusion

The results of this study indicate that the structure of the original version of the MBFES mean it is a valid and reliable tool for evaluating maternal perceptions of the breastfeeding experience in Spain. This scale provides a new outcome variable for the breastfeeding process. Based on the mother’s own perception, it complements the duration of breastfeeding variable. Finally, we conclude that satisfaction with breastfeeding is negatively associated with perceived difficulty in maintaining breastfeeding while working and positively associated with the rate of breastfeeding at five months postpartum. The scale can also be used as an indicator of successful breastfeeding outcomes.

A number of implications for the use of the scale and recommendations for future lines of research emerge from this paper. From a research perspective, we recommend analyzing the responsiveness of the scale (useful for determining the effectiveness of interventions and programs) and identifying and controlling for various biases, such as common method bias. Furthermore, validation in other samples, such as those from vulnerable socioeconomic backgrounds, would be desirable, as previous validation studies have been performed in women with high levels of both income and education. From the perspective of healthcare professionals, this study provides a tool with good psychometric properties, capable of capturing the holistic experience of breastfeeding and predicting breastfeeding. This information will be useful for the development of breastfeeding promotion and support interventions. In terms of policy, this research could provide policymakers with a valuable tool for assessing the quality of breastfeeding, which may help measure the effectiveness of breastfeeding promotion strategies.

### Electronic supplementary material

Below is the link to the electronic supplementary material.

Supplementary Material 1

Supplementary Material 2

Supplementary Material 3

## Data Availability

The datasets used and analysed during the current study are available from the corresponding author on reasonable request.
