# Supplementary figures and images for: Psychometric properties of the maternal breastfeeding evaluation scale: a confirmatory factor analysis
Source: BMC Pregnancy Childbirth. 2024 Jul 18;24:486. doi: 10.1186/s12884-024-06693-8 (PMC11264472; doi:10.1186/s12884-024-06693-8)

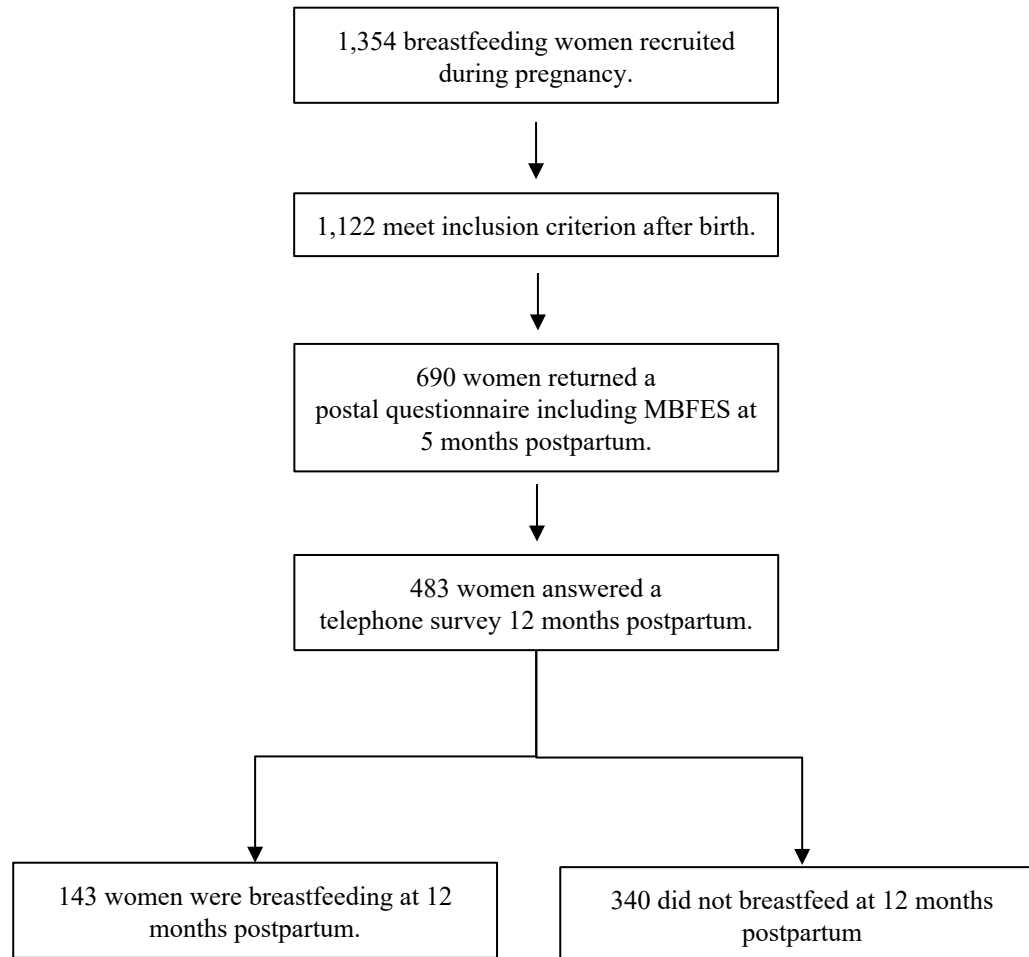

Supplement: Supplementary file 1 — Supplementary Material 1 [file 12884_2024_6693_MOESM1_ESM.pdf]
