# Supplementary material for: Psychometric properties of the maternal breastfeeding evaluation scale: a confirmatory factor analysis
Source: BMC Pregnancy Childbirth. 2024 Jul 18;24:486. doi: 10.1186/s12884-024-06693-8 (PMC11264472; doi:10.1186/s12884-024-06693-8)
Supplement: Supplementary file 2 — Supplementary Material 2 [file 12884_2024_6693_MOESM2_ESM.docx]

**Appendix 2.** Description of different versions of the Maternal Breastfeeding Evaluation Scale.

|  | **Sample** | **Language** | **Year** | **Nº items** | **Construct Validity** | **Internal consistency** | **Criterial validity** |
| --- | --- | --- | --- | --- | --- | --- | --- |
| **MBFES [13]** | 442 mother who had given birth during the previous year and had breastefed.  EEUU. | English | 1994 | 30 items | EFA  1. **Maternal enjoyment / role attainment** (14 items:1, 2, 6 ,9 ,11 ,12 ,16 ,17 ,18 ,20 ,21 ,23 ,25 and 30)  **2. Infant** **satisfaction/growth** (8 items: 3R, 4, 7R, 10, 15R, 19R, 24, 28R)  3. **Lifestyle / maternal body image** (8 items: 5R, 8R, 13, 14R, 22R, 26, 27R and 29R) | **Total test:** α = 0.93  **Maternal enjoyment / role attainment:** α= 0.93  **Infant satisfaction/growth:** α= 0.88  **Lifestyle / maternal body image:** α= 0.80 | Correlation with breastefeding and duration of breastfeeding (concurrent criterion validity)  **Total test:** r=0.83; r=0.48  **Maternal enjoyment / role attainment:** r=0.79; r=0.44  **Infant satisfaction/growth:** r=0.70; r=0.45  **Lifestyle / maternal body image:** r=0.55; r=0.28  All correlations p<0.001. |
| **MFBES-A [16]** | 450 mothers  Líbano | Arabic | 2021 | 26 items | EFA  1. **Maternal enjoyment / role attainment** (10 items:1, 2, 6 ,9 ,12 ,17, 20, 21, 23, 25 and 30)  **2. Infant** **satisfaction/growth** (9 items: 3R, 4, 7R, 10, 11, 16, 18, 24 and 28R)  3. **Lifestyle / maternal body image** (6 items: 5R, 8R, 14R, 22R, 26 and 27R) | **Total test:** α = 0.89  **Maternal enjoyment / role attainment:** α= 0.87  **Infant satisfaction/growth:** α= 0.88  **Lifestyle / maternal body image:** α= 0.68 | Correlations with exclusive breastefeeding at 1 and 3 months postpartum. El MBFES was administered at 1 month.  **Total test:** r  r=0.27 (p<0.001);  r=0.26 (p<0.001)  **Maternal enjoyment / role attainment:**  r=0.22 (p<0.001); r=0.22 (p<0.001)  **Infant satisfaction/growth:** r=0.37 (p<0.001); r=0.31 (p<0.001)  **Lifestyle / maternal body image:**  r=0.01 (p=0.797); r=0.06 (p=0.248) |
| **JMBFES [24]** | 414 mothers  Japan | Japanese | 2013 | 23 items | EFA  **1. Maternal satisfaction** (11 items: 1, 2, 6, 9, 12, 17, 18, 20, 21, 23 y 30  **2. Perceived benefit to baby** (7 items: 4, 7, 10, 11, 16, 24 y 25)  **3. Potentially negative aspects** (5 items: 8R, 14R, 22R, 27R and 29R)  *Item 7 is understood as a positive characteristic unlike the original.* | **Total test:** α = .77  **Maternal satisfaction:** α= 0.91  **Perceived benefit to baby:** α= 0.84  **Potentially negative aspects:** α= 0.77 | Multiple linear regression analysis with intention to breastfeed (prenatal) and breastefeeding (1 month posnatal, 4 months posntatal). At 4 months, questions were asked retrospectively about the intention and about breastfeeding during the month.  **Total test:**  Coef=7.90; p<0.001; Coef=6.94; p<0.001; Coef=6.72; p<0.001.  **Maternal satisfaction:** Coef=3.76; p<0.001; Coef=2.49; p<0.001; Coef=1.69; p=0.01  **Perceived benefit to baby:**  Coef=2.56; p<0.001; Coef=3.60; p<0.001; Coef=3.31; p<0.001  **Potencially negative:** Coef=1.30; p=0.005; Coef=0.75; p=0.08; Coef=1.42; p=0.001 |
| **Brazilian version of MBFES [23]** | 287 mothers 30 days after childbirth  Brazil | Portuguese | 2020 | 29 items | EFA  1. **Pleasure and fulfillment of the maternal role** (14 items:1, 2, 6 ,9, 11, 12, 17,18, 20, 21, 23 and 30)  **2. Child´s growth, development and satisfaction** (8 items: 3R, 4, 7R, 10, 16, 19R, 24, 25, 28R)  3. **Maternal physical, emotional and social aspects** (7 items: 5R, 8R, 13, 14R, 15, 22R, 26 and 29R) | **Total test:** α= 0.88  **Pleasure and fulfillment of the maternal role:** α= 0.92  **Child´s growth, development and satisfaction:** α= 0.75  **Maternal physical, emotional and social aspects:** α= 0.70 | Concurrent criterial validity of total punctuation of the scale  Breastfeeding at 30 days  Yes (M=121.75; SD=12.92)  No (M=101.91; SD=22.57) (p<0.001)  Intention regarding breastfeeding duration  >12 months (M=125.30; SD=13.67)  <12 months (M=117.29; SD=16.31)  (p<0.002) |
| **MBFES-E [25]** | 136 madres at 5 months postpartum  Spain | Spanish | 2021 | 30 items | EFA  1. **Maternal enjoyment / role attainment** (16 items:1, 2, 4 ,6 ,9 ,11 ,12 ,16 ,17, 18, 20, 21, 23, 25, 26 and 30)  **2. Infant** **satisfaction/growth** (8 items: 3R, 7, 10, 13, 15R, 19R, 24, 28R)  3. **Lifestyle / maternal body image** (6 items: 5R, 8R, 14R, 22R, 27R, 29R)  *Item 7 is understood as a positive characteristic unlike the original.* | **Total test:** α = 0.93  **Maternal enjoyment / role attainment:** α= 0.92  **Infant satisfaction/growth:** α= 0.92  **Lifestyle / maternal body image:** α= 0.93 | Not analyzed. |

**Note:** items marked with R need to be reversed.
