# Supplementary material for: Psychometric properties of the maternal breastfeeding evaluation scale: a confirmatory factor analysis
Source: BMC Pregnancy Childbirth. 2024 Jul 18;24:486. doi: 10.1186/s12884-024-06693-8 (PMC11264472; doi:10.1186/s12884-024-06693-8)
Supplement: Supplementary file 3 — Supplementary Material 3 [file 12884_2024_6693_MOESM3_ESM.docx]

**Table 3.** Performance of the MBFES scale item.

|  | **M (SD)** | **Range**  **(Min-Max)** | **Floor effect**  **n (%)** | **Ceiling effect**  **n (%)** |
| --- | --- | --- | --- | --- |
| I1 | 4.75 (0.66) | 1-5 | 6 (0.85) | 590 (83.69) |
| I2 | 4.71 (0.72) | 1-5 | 7 (0.99) | 576 (81.70) |
| I3R | 4.58 (0.97) | 1-5 | 26 (3.69) | 559 (79.29) |
| I4 | 4.55 (0.98) | 1-5 | 23 (3.26) | 539 (76.45) |
| I5R | 4.15 (1.13) | 1-5 | 24 (3.40) | 381 (54.04) |
| I6 | 4.71 (0.72) | 1-5 | 8 (1.34) | 575 (81.56) |
| I7 | 3.87 (1.22) | 1-5 | 44 (6.24) | 292 (41.42) |
| I8R | 3.37 (1.26) | 1-5 | 63 (8.93) | 159 (22.55) |
| I9 | 4.40 (0.88) | 1-5 | 10 (1.41) | 422 (59.86) |
| I10 | 4.31 (1.10) | 1-5 | 28 (3.97) | 446 (63.26) |
| I11 | 4.30 (1.06) | 1-5 | 25 (3.55) | 422 (59.86) |
| I12 | 4.58 (0.84) | 1-5 | 11 (1.56) | 520 (73.76) |
| I13 | 3.78 (1.04) | 1-5 | 26 (3.69) | 214 (30.35) |
| I14R | 3.48 (1.25) | 1-5 | 44 (6.24) | 190 (26.59) |
| I15R | 2.36 (1.34) | 1-5 | 239 (33.90) | 75 (10.64) |
| I16 | 4.28 (1.05) | 1-5 | 28 (3.97) | 404 (57.30) |
| I17 | 4.14 (0.98) | 1-5 | 16 (2.26) | 316 (44.82) |
| I18 | 4.53 (0.82) | 1-5 | 9 (1.27) | 482 (68.37) |
| I19R | 3.73 (1.52) | 1-5 | 101 (14.33) | 348 (49.36) |
| I20 | 4.19 (1.05) | 1-5 | 23 (3.26) | 373 (52.91) |
| I21 | 4.34 (1.01) | 1-5 | 26 (3.69) | 426 (60.43) |
| I22R | 3.40 (1.31) | 1-5 | 76 (10.78) | 191 (27.09) |
| I23 | 4.00 (1.11) | 1-5 | 31 (4.40) | 304 (43.12) |
| I24 | 4.10 (1.23) | 1-5 | 43 (6.10) | 386 (54.75) |
| I25 | 3.98 (1.07) | 1-5 | 26 (3.69) | 294 (41.70) |
| I26 | 3.62 (1.23) | 1-5 | 42 (5.96) | 216 (30.64) |
| I27R | 4.38 (0.95) | 1-5 | 12 (1.70) | 442 (62.70) |
| I28R | 4.30 (1.11) | 1-5 | 35 (4.96) | 438 (62.13) |
| I29 | 3.95 (1.16) | 1-5 | 23 (4.68) | 335 (42.55) |
| I30 | 4.15 (1.02) | 1-5 | 23 (3.26) | 335 (47.52) |
| Maternal enjoyment/role attainment | 69.23 (10.75) | 19-80 | - | - |
| Infant satisfaction/growth | 31.03 (6.14) | 8-40 | - | - |
| Lifestyle/maternal body image | 22.73 (4.79) | 6-30 | - | - |

**Note:** items marked with R need to be reversed.
